# A comparison between health research output and burden of disease in Arab countries: evidence from Palestine

**DOI:** 10.1186/s12961-018-0302-4

**Published:** 2018-03-15

**Authors:** Loai Albarqouni, Khamis Elessi, Niveen M. E. Abu-Rmeileh

**Affiliations:** 1 Centre for Research in Evidence Based Practice (CREBP), Faculty of Health Science and Medicine, Bond University, Robina, QLD Australia; 2 Evidence-Based Medicine Unit, Faculty of Medicine, Islamic University, Gaza, Palestine; 3 Institute of Community and Public Health, Birzeit University, Ramallah, West Bank, Palestine

**Keywords:** Health systems research, research agenda, priority-setting, observatory, research and development

## Abstract

**Background:**

Research conducted on conditions responsible for the greatest disease burden should be given the highest priority, particularly in resource-limited settings. The present study aimed to assess the research output in relation to disease burden in Palestine and to identify the conditions which are under- or over-investigated, if any.

**Methods:**

We searched PubMed and Scopus for reports of original research relevant to human health or healthcare authored by researchers affiliated with Palestinian institutions and published between January 2000 and December 2015. We categorised the condition studied in included articles using the Global Burden of Disease (GBD) taxonomy. Data regarding burden of disease (percentage of deaths and disability-adjusted life years (DALYs)) was obtained from the Palestine profile in the GBD study. We examined the degree of discordance between the observed number of published articles for each disease/condition with the expected number based on the proportion of disease burden of that disease/condition.

**Results:**

Our search identified 2469 potentially relevant records, from which 1650 were excluded following the screening of titles and abstracts. Of the remaining 819 full-text articles, we included 511 in our review. Communicable (infectious) diseases (*n* = 103; 20%) was the condition with the highest number of published studies. However, cancer (*n* = 15; 3%) and chronic respiratory diseases (*n* = 15; 3%) were the conditions with the lowest number of published studies. Research output was poorly associated with disease burden, irrespective of whether it was measured in terms of DALYs (rho = −0.116, *P* = 0.7) or death (rho = 0.217, *P* = 0.5). Cardiovascular disease, cancer, and maternal and neonatal deaths accounted for more than two-thirds of the total deaths in Palestine (67%), but were infrequently addressed (23%) in published articles.

**Conclusions:**

There is evidence of research waste measured by a mismatch between the health burden of certain diseases/conditions and the number of published research reports on those diseases/conditions in Palestine. A national research priority-setting agenda should be developed to meet the local community’s need for quality evidence to implement independent and informed health policies.

## Background

Palestine, a Middle East and North Africa (MENA) region country, consists of the West Bank, including East Jerusalem, and the Gaza Strip [1]. It has a population of 4.8 million, 42% of whom are registered refugees – indeed, 26% and 68% of those living in the West Bank and Gaza, respectively, are refugees [2]. Over the past decades, Palestinians have undergone a rapid epidemiological transition characterised by a growing burden of non-communicable diseases (NCDs) such as cardiovascular disease (CVD), diabetes and cancer [3].

Palestine, as elsewhere, is facing scarce financial resources and limited research infrastructure, impeding the allocation of adequate resources to health research development [4]. Thus, it is of paramount importance to maximise the utilisation of the, albeit inadequate, resources allocated to health research by prioritising the necessary evidence required by the Palestinian community to develop independent and informed health policies.

Substantial gaps exist between the health research that is needed and that which is conducted, indicating a lack of appropriate prioritisation of health research [5]. In their 2009 *Lancet* seminal report ‘*Avoidable waste in the production and reporting of research evidence*’ [6], Chalmers and Glasziou noted that the dramatic mismatch between questions addressed by researchers and questions of relevance to the community is one of the factors contributing to the estimated 85% waste in health and medical research. Research prioritisation is therefore required to guide resource allocation to areas of highest priority and to strengthen the links between research, policy and action [7]. There is a need for an accountable, transparent and sustainable approach for research prioritisation on the basis of societal needs (e.g. disease burden) [8]. The burden of disease is a measure that represents the relative impact of different diseases and conditions on population health, and is frequently measured by two indices – mortality (i.e. total deaths) and morbidity (i.e. disability-adjusted life years (DALYs)).

The highest priority should be given to research conducted on conditions that most substantially contribute to disease burden. Topic coverage in research output may serve as a proxy of research prioritisation, with imbalances between the burden of disease and the research output likely indicating specific diseases/conditions that are relatively under- or over-investigated compared to their attributable burden. Highlighting this imbalance is therefore critical in providing guidance to stakeholders/policy-makers on the allocation of limited resources in health research. A recent analysis showed a weak association between the global burden of disease and the number of published randomised trials [9]. However, there are no previous studies examining the association between burden of disease and research output in Palestine or in the MENA region. This information can inform policy-makers on the gaps between health research needs and research conducted in Palestine, with implications on research prioritisation, funding allocations and research agenda-setting in Palestine. In this study, we compared the distribution of the output of published health and medical research from Palestine and the distribution of the burden of disease in Palestine and investigated whether specific conditions/diseases are under- or over-investigated.

## Methods

We used the same dataset as in a previous analysis that aimed to assess the quality of reporting of Palestinian medical and health research, but we updated the search till the end of 2015 [10].

### Data sources and search strategy

We searched PubMed and Scopus databases for articles of Palestinian medical and public health studies published between January 1, 2000, and December 31, 2015. We searched for the following terms in the author affiliation, title or abstract: ‘palestin*’, ‘jerusalem’, ‘west bank’, ‘gaza’, ‘oPt’, or ‘occupied Palestinian territories’. No language restrictions were applied.

### Eligibility criteria

We included all articles of original research authored and/or co-authored by researchers affiliated with Palestinian institutions. We included only articles that reported medical and health studies. We included quantitative studies, whether observational (cross-sectional, cohort, case–control studies, and case series and reports), interventional (controlled trials) or systematic reviews and meta-analyses. We excluded studies where none of the authors was affiliated with a Palestinian institution, even if they included Palestinian participants. We also excluded studies not involving humans as well as studies on health systems research.

### Study selection

Two reviewers independently screened the titles and abstracts of retrieved records against our eligibility criteria. The same two reviewers independently assessed the full texts of all potentially eligible articles. Disagreements between reviewers were resolved by discussion and consensus. Reasons for exclusion were identified and documented.

### Data collection

Two reviewers independently and in duplicate used a data extraction form to extract the required data from each article, including (1) bibliographic details – authors, journal name, and year of publication; (2) number of authors, the first author’s affiliation and any national, regional (i.e. MENA region) or international collaboration; (3) description of disease or condition evaluated according to groups of global burden of disease [11] (in studies tackling more than one disease, we considered the disease relevant to the primary objective of the study, if not reported, then the most highlighted disease throughout the article); (4) sample size (in case of a systematic review and/or meta-analysis, we considered the number of included studies as the sample size); (5) type of study question (prevalence, etiological, diagnostic, prognostic, interventional) and study design (systematic review, randomised controlled trials (RCT), non-randomised intervention study (e.g. controlled trials), cohort, case–control, cross-sectional and case report/series) [12]; and (6) source of funding (international, local government, academic institution or industry, and not reported/unclear).

#### Burden of disease

Data on the burden of disease in Palestine were obtained from the Global Burden of Disease (GBD) 2015 study [13]. GBD is a systematic evaluation and quantification of the burden of major diseases and conditions categorised by age, sex and country. Burden is estimated using both deaths and DALYs [11]. We obtained country-specific estimates of the total number of DALYs and deaths in Palestine for each disease/condition, with death defined as the number of deaths due to a specific cause (disease or injury) within the total population. DALYs is a standardised metric that reflects the discrepancy between existing and ideal health status. DALYs account for both years of life lost due to premature death and years lived with disability.

### Data analyses and interpretation

Descriptive analysis was performed using frequency and percentages. We plotted the total number of research articles for each disease/condition category against their associated deaths (number of deaths per 100,000 population) and DALYs (number of DALYs per 100,000 population). We assessed the correlation between research output and burden of disease using the Spearman correlation test (i.e. Spearman’s rho coefficient). We also assessed whether the distribution of research output has matched the distribution of disease burden (i.e. in terms of both deaths and DALYs) for each disease/condition, by examining the degree of discordance between the observed number of published articles for each disease/condition with the expected/proportionate number of published articles based on the proportion of disease burden for that disease/condition (e.g. the expected number of published articles for CVD = % of burden caused by CVD × total published reports = 39.3% × 511 = 201). The difference between observed and expected number of published articles is expected to be zero when there is a good alignment between research output and disease burden. A positive difference indicates that the disease/condition was over-studied (i.e. research surplus), while a negative difference suggests that the disease/condition was under-studied (i.e. research deficit). We used Microsoft Excel and R software (version 3.2.3) for data preparation and analysis.

## Results

**Fig. 1:**
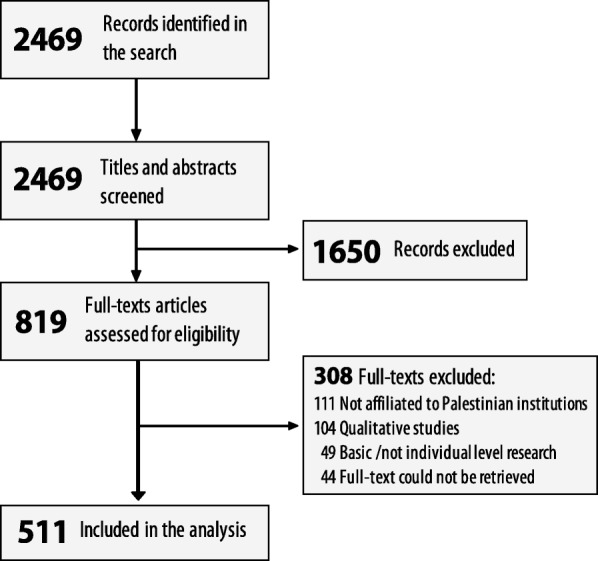
PRISMA Flowchart of the study selection process

Our search identified 2469 potentially relevant records, from which 1650 were excluded based on screening titles and abstracts. Of the remaining 819 full-text articles, we included 511 in our review. The reasons for excluding the 308 articles were (1) none of the authors/co-authors was affiliated to a Palestinian institution (36%); (2) it was an article of qualitative research (34%); (3) it had not been conducted on individual humans (e.g. basic science research) (16%); and (4) full texts could not be retrieved (14%) (Fig. 1).

### Characteristics of included studies

First authors of 361 (71%) articles were affiliated with Palestinian institutions, 35 (7%) with regional institutions, and 115 (23%) with international institutions. Of the 511 included articles, 152 (30%) involved collaboration between more than one Palestinian institution, 31 (6%) between Palestinian and regional institutions, and 240 (47%) between Palestinian and international institutions. Most (371; 73%) of the articles were co-authored by 2–6 authors.

The number of published Palestinian medical and health research articles increased over time, almost doubling every 5 years (13% between 2000 and 2004, 32% between 2005 and 2010, and 55% between 2011 and 2015).

Table 1 provides information on the general characteristics of the included articles. The most frequent study design was cross-sectional studies (*n* = 381; 75%), while 16 (3%) were controlled trials, and only 4 (0.8%) were systematic reviews. Most of the articles (368; 72%) presented prevalence/association studies, 61 (12%) therapeutic studies, 27 (5%) aetiological studies, 17 (3%) prognostic studies, and 11 (2%) diagnostic studies. The majority of included articles had a sample size of less than 200 participants (*n* = 406; 80%). Sources of funding were not mentioned in approximately two-thirds (351; 68.7%) of the articles. Of those reporting, over two-thirds were international (110; 21.5%).

**Table 1:** Characteristics of included studies (*n* = 511)

	No. of studies (%)
Total	511 (100)
Affiliation(s) of the first author	
Palestine	361 (70.6)
Regional	35 (6.9)
International	115 (22.5)
Collaborations	
The same Palestinian institution	88 (17.2)
Different Palestinians Institutions	152 (29.7)
Palestinian and Regional Institutions	31 (6.1)
Palestinian and International institutions	240 (47.0)
Number of authors per article	
1	53 (10.4)
2–6	371 (72.6)
> 7	87 (17.1)
Publication year	
2000–2004	68 (13.3)
2005–2010	164 (32.1)
2011–2015	265 (54.6)
Sample size (No. of participants)	
< 50	187 (36.6)
50–200	219 (42.9)
> 200	105 (20.5)
Type of the study question	
Prevalence/association	368 (72.0)
Aetiology/risk factors	27 (5.3)
Diagnosis	11 (2.2)
Therapeutic/intervention	61 (11.9)
Prognosis	17 (3.3)
Others (case report)	27 (5.3)
Study design	
Cross-sectional	381 (74.6)
Systematic review	4 (0.8)
Randomised controlled studies	8 (1.6)
Non-randomised interventional studies	8 (1.6)
Cohort	38 (7.4)
Case–control	45 (8.8)
Case reports/series	27 (5.3)
Funding sources	
Not reported/unclear	351 (68.7)
Local (governmental/industry/institutional)	50 (9.8)
International	110 (21.5)

### The distribution of research output and disease burden by disease/condition categories

The distribution of Palestinian health research output and disease burden (DALYs and deaths) across various diseases/conditions is presented in Table 2. Communicable (infectious) diseases (*n* = 103; 20%), nutritional diseases (*n* = 55; 11%), and mental and substance use disorders (*n* = 54; 11%) were the conditions with the highest number of published studies. However, cancer (*n* = 15; 3%), chronic respiratory (*n* = 15; 3%), and gastrointestinal diseases (*n* = 16; 3%) were the conditions with the lowest number of published studies.

**Table 2:** The distribution of research output and disease burden (DALY and death) per disease category

Disease/Condition	Research output (% total)	Rank	Number of involved participants	Rank	DALY rate per 100,000 population (% total DALY)^a,b^	Rank	Death rate per 100,000 population (% total death)^a,c^	Rank	DALY per 100,000 population per study^a,d^	Death per 100,000 population per study^a,e^
Infectious	103 (20)	1	18,101	1	1170 (5)	7	18 (6)	6	11.4	0.2
Nutritional	55 (11)	2	15,450	2	536 (3)	11	0.1 (0)	12	9.7	0
Mental	54 (11)	3	13,009	3	2163 (10)	5	1.2 (0)	11	40.1	0
Cardiovascular	52 (10)	4	9598	5	3221 (15)	2	124 (39)	1	61.9	2.4
Maternal, neonatal and congenital	50 (10)	5	6763	6	5578 (26)	1	48 (15)	2	111.6	1
Blood, urogenital and endocrine diseases	48 (9)	6	6629	7	1004 (5)	8	19 (6)	5	20.9	0.4
Musculoskeletal and neurological	37 (7)	7	1744	10	2449 (11)	3	12 (4)	7	66.2	0.3
Diabetes	36 (7)	8	11,157	4	559 (3)	9	9 (3)	9	15.5	0.2
Injuries	30 (6)	9	5779	8	2314 (11)	4	28 (9)	4	77.1	0.9
Gastrointestinal	16 (3)	10	1454	11	350 (2)	12	8 (3)	10	21.9	0.5
Cancer	15 (3)	11	864	12	1420 (7)	6	40 (13)	3	94.7	2.7
Chronic respiratory	15 (3)	12	2559	9	541 (3)	10	9 (3)	8	36.1	0.6

^a^Data on DALYs and death in Palestine were obtained from the Global Burden of Disease (GBD) 2015 study [13]

^b^% total DALY = (DALY rate per 100,000 population for a specific disease or condition ÷ DALY rate per 100,000 population) × 100; larger value denotes higher contribution of this disease/condition to the total DALY in Palestine

^c^% total death = (death rate per 100,000 population for a specific disease or condition ÷ death rate per 100,000 population) × 100; larger value denotes higher contribution of this disease/condition to the total deaths in Palestine

^d^DALYs per 100,000 population per study = (DALY per 100,000 population for a specific disease or condition ÷ research output for the same disease or condition) × 100

^e^Deaths per 100,000 population per study = (death per 100,000 population for a specific disease or condition ÷ research output for the same disease or condition) × 100

Maternal, neonatal and congenital conditions were the main cause of DALYs accounting for 26% of the total DALYs in Palestine in 2015, followed by cardiovascular (15%), musculoskeletal and neurological conditions (11%). CVD, maternal, neonatal and congenital conditions, and cancer were the main causes of death accounting for 39%, 15%, and 13% of the total deaths in Palestine in 2015.

### Association between research output and disease burden

**Fig. 2:**
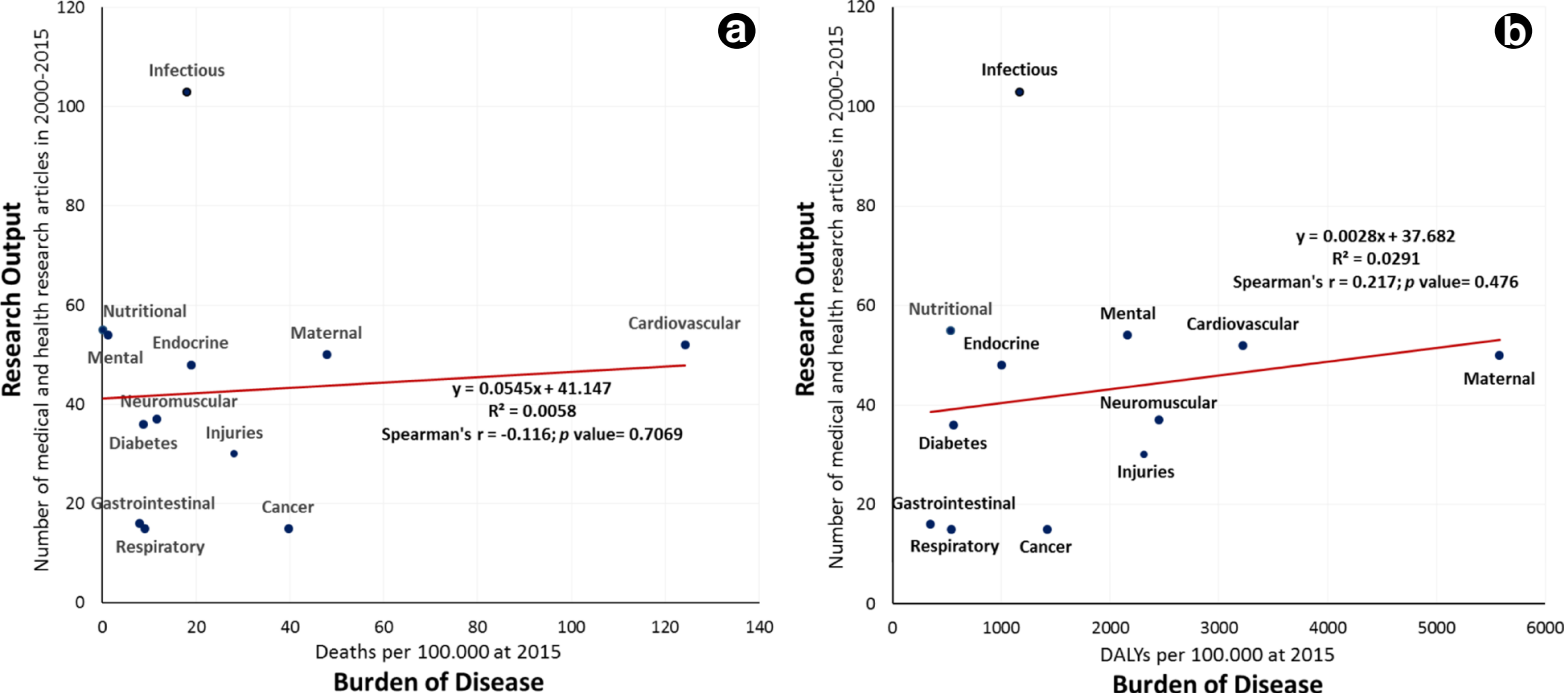
Relationship between research output (i.e. number of Palestinian medical and health research articles research published between 2000 and 15) and burden of disease as the number of deaths up to 2015 (**a**) or number of disease-adjusted life years (DALYs) up to 2015 (**b**)

Cancer, CVD, and maternal, neonatal and congenital conditions were all understudied since they have the highest ratios of disease burden to research output (DALYs per 100,000 population per study: 94.7, 61.9 and 111.6, respectively; deaths 100,000 population per study: 2.7, 2.4 and 1, respectively). However, nutritional, infectious, and mental and substance use disorders were all over-studied with regards to their contribution to the disease burden (DALYs 100,000 population per study: 9.7, 11.4 and 40.1, respectively; deaths 100,000 population per study: 0, 0.2 and 0, respectively) (Table 2). Figure 2 shows that research output was poorly correlated with disease burden, irrespective of whether measured in terms of DALYs (rho = −0.116, *P* = 0.7) or death (rho = 0.217, *P* = 0.5).

### Expected versus observed research output

**Fig. 3:**
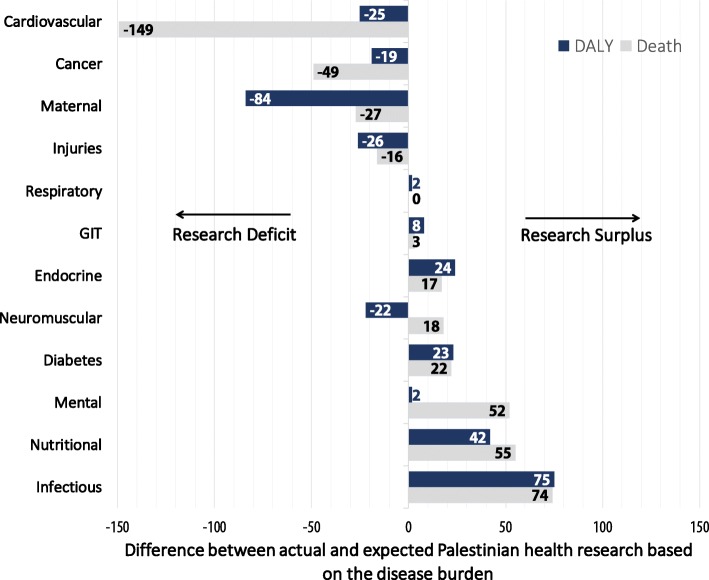
Difference between the actual observed number of published Palestinian health research articles for each disease/condition and expected number in proportion to disease burden

To further assess the association/proportionality between research output and disease burden, we examined the actual observed and expected number of studies as a function of the disease burden (Fig. 3). The difference between the actual and expected number of articles demonstrates whether each condition/disease was under- or over-represented in published Palestinian research articles (0: matched/balanced, negative: understudies/represented: positive: over-studied/represented). Cancer, musculoskeletal diseases, CVD and diabetes were the most understudied conditions relative to their burden, while infectious and nutritional conditions were the most over-studied (Fig. 3).

## Discussions

Our study indicates that there is an evident mismatch between the distribution of disease burden and conditions/diseases that were investigated in published articles from Palestine between 2000 and 2015. It is worrisome that CVD, maternal and neonatal diseases, and cancer account for more than two-thirds of the total deaths in Palestine (67%), but are infrequently addressed (23%) in published articles.

Our findings are in line with a previous investigation of research output (i.e. 66 RCTs published in five leading medical journals) in Latin America that found a poor correlation between disease burden and research output [14]. This was also evident in a recent cross-sectional analysis of 1097 RCTs [9], which found that global burden of disease is poorly associated with the number of published randomised trials (Spearman’s *r* = 0.35; *P* < 0.001) as well as with the number of recruited participants (Spearman’s *r* = 0.33; *P* < 0.001). Similarly, an analysis of a random sample of 2381 records of trials registered in the WHO’s International Clinical Trials Registry Platform (ICTRP), found that there is little correlation between disease burden and the global distribution of registered clinical trials, with research and development not adequately meeting the needs of populations in lower-income countries [15]. A 2002 study of 1179 published randomised trials from sub-Saharan Africa (48% from South Africa) showed a good correlation between the estimated burden of disease and the number of trials performed (Spearman’s *r* = 0.53, *P* = 0.024) and the number of participants randomised (Spearman’s *r* = 0.68, *P* = 0.002) [16]. However, a recent sub-set analysis of all these RCTs found that a very poor correlation between disease burden and the number of trials (Spearman’s *r* = 0.17) [9]. This can be explained by the rapid epidemiological transition in sub-Saharan Africa, involving an increase in NCD burden, and yet a prioritisation of communicable diseases (e.g. HIV and malaria) in sub-Saharan countries’ health research systems and capacity [17, 18].

A recent scoping review of 3776 NCD-related reports published between 2000 and 2013 from seven Arab countries [19], found a mismatch between cause-specific death rates and research output, with a relative surplus of reports on cancer and a relative deficit of those on CVDs. The subset analysis of reports from Palestine showed a deficit of studies on both CVDs and cancer. However, this study is limited to publications related to NCDs, to the exclusion of other diseases/conditions.

Previous investigations have also shown that there is a mismatch between disease burden and allocated funds. An analysis of the relation between WHO’s budgetary allocations and burden of disease found a misalignment between fund allocation and disease burden, with a noticeable skew towards infectious diseases [20]. However, Gross et al. [21] compared the estimates of National Institute of Health disease-specific funding in 1996 with the burden of disease, and found that funding was more strongly associated with DALYs (*r* = 0.62, *P* < 0.001) than the number of deaths (*r* = 0.40, *P* = 0.03).

Important to note is the slight discrepancies between the findings related to comparing research output to death rates and DALYs, respectively. While high case fatality rates for some conditions may inflate death rates (e.g. CVD and cancer), disabling conditions with no cure and long duration (e.g. musculoskeletal and neurological) inflate DALYs. For instance, we found that ‘Musculoskeletal and Neurological’ and ‘Injuries’ conditions were understudied when considering DALYs but less so when considering death rates.

The observed discordance between the focus of the published research from Palestine and disease burden can be explained by several factors, including (1) researchers’ interest and expertise; (2) limited research infrastructure and funding resources, which drive researchers to accept the funder agenda, not commonly aligned with local community needs [22–24]; and (3) lack of communication between policy-makers and researchers to agree on national research priorities [20, 25].

A limitation to this review is that we only searched two databases (PubMed and Scopus) and we may have therefore missed a number of relevant studies not indexed therein, as well as any unpublished studies. Similarly, excluding lab-based studies may have influenced the results of our study (e.g. cancer studies are frequently lab-based, and therefore more likely missed due to our eligibility criteria). Further, there is some degree of subjectivity in assigning each study to a particular disease or condition. However, two reviewers assigned studies to each category independently and in duplicate. Important to note is that published research output may not necessarily accurately reflect all work being conducted in a particular condition/disease area. Indeed, research output is only one of five core indicators of potential societal benefits of health research, as described by Hanney et al. [26] in their Payback model (i.e. a framework of five core indicators: knowledge production, research targeting and capacity, informing policy, health and health sector benefits, and economic benefits). We also found that the clear majority (96%) of health research from Palestine were observational studies, which have limited potential to inform health policy-making compared to a higher level of evidence (e.g. systematic reviews and RCTs).

### Implications of our findings

#### Establish a national medical and health research priority-setting in Palestine

Priority-setting is essential for efficient use of limited resources, and an integral step needed in the national research management process to assist the allocation of limited resources to meet national health goals. Otherwise, there is a risk that research topics are determined and imposed by funding organisations for their own agenda and policies.

National research priority-setting should be derived using transparent methodologies, including an evidence-based systematic assessment and situation analysis. Stakeholder involvement in the priority-setting exercise should be inclusive to ensure the extensive participation of researchers, clinicians, university research boards, government, funders, civil organisations, industry, patients and the public. Crowe et al. [27] found a persistent mismatch between patients’, clinicians’ and the research communities’ priorities. In a recent comprehensive assessment of health research priority-setting initiatives in developing countries, McGregor et al. [25] found that the majority of the 91 identified priority-setting initiatives took place at the global level (i.e. those with global health agenda such as eradicating specific diseases) with a developing country focus. However, most did not have any evidence of implementation or follow-up.

#### Enhance the capacity of national researchers and urge them to conduct prioritised research

Lack of sufficient health research capacity is still a major barrier to conduct evidence-based health research to inform policy and improve health [28]. The World Health Report 2013 focused on the importance of all nations being producers and consumers of research (i.e. to develop a capacity to not just adopt the evidence, but to adapt it to local circumstances [29]). Therefore, research capacity-building should be given a higher priority and needs to be equally valued as research outputs by development parties [30, 31].

#### Foster dialogue between researchers, policy-makers, funders and end-users/patients

Communication between researchers, academics, decision-makers and patients/public should be started as early as possible during the research priority-setting and continued throughout the research conduction. Further, results dissemination should be fostered by knowledge translation interventions to enhance the uptake of evidence into practice. This exchange of ideas and information will facilitate the bridging of the gap between research conducted at research institutions and the community’s and decision-makers’ needs.

## Conclusions

Despite the encouraging increase in the research output from Palestine over the last decades, there remains a weak association between research output and burden of disease. NCDs (e.g. CVD and cancer) receive much less attention by researchers despite accounting for most of the disease burden in Palestine. National research priority-setting should be developed to meet the Palestinian community’s need for quality evidence to establish independent and informed health policies.
